# Campbell Title Registrations to Date – May 2025, and Discontinued Protocols

**DOI:** 10.1002/cl2.70052

**Published:** 2025-06-02

Details of new titles for systematic reviews or evidence and gap maps that have been accepted by the Editor of a Campbell Coordinating Group are published in each issue of the journal. If you would like to receive a copy of the approved title registration form, please send an email to the Managing Editor of the relevant Coordinating Group.

A list of discontinued protocols appears below these new titles. If you are interested in continuing a project, please get in touch with the Managing Editor of the relevant Coordinating Group or email info@campbellcollaboration.org.

## 1 Ageing

The effectiveness of interventions in preventing or managing post‐stroke cognitive impairments and enhancing the quality of life for stroke survivors: A protocol for systematic review of randomised controlled trials.

Memory Lucy Mtambo, Tetisya Ragunathan, Adeola Folayan, Kia Fatt Quek.

31 March 2025.

## 2 Children and Young Persons Wellbeing

The Global Commercialization of Human Milk for Infant Consumption: A scoping review.

Heather Rusi, Jennifer Dion, Smitha Rodrigues, Merilee (Meredith) Brockway.

23 April 2025.

Exploring of Circadian Misalignments in Eating and Sleep on Health and School Performance in Children and Adolescents: A scoping review protocol.

Nur'Ain Mardhiyah Harun, Fatin Hanani Mazri, Zahara Abdul Manaf, Arimi Fitri Mat Ludin, Haslina Abdul Hamid, Nik Nur Izzati Nik Mohd Fakhruddin.

23 April 2025.

## 3 Disability

Access to financial resources to people with disabilities for entrepreneurship: A systematic review protocol.

Gamariel Mboya, Jackline Kiwelu, Gadiel Ketto, Howard Omukami.

27 March 2025.

Context, mechanisms and outcomes of reasonable adjustments across acute hospitals for people with intellectual disability: A scoping review protocol.

Mairead Moloney, Laurence Taggart, Owen Doody.

21 March 2025.

## 4 Social Welfare

Telehealth services for outpatient mental health in rural populations: A systematic review.

Hannah Shanks, Julie Birkenmaier, Brandy Maynard.

31 March 2025.

Understanding the health care experiences of women who use illegal substances: An intersectional scoping review.

Marjan Kekanovich, Tyler Glass, Amanda Ross‐White, Danielle Macdonald, Susan Bartels, Jacqueline Galica.

18 May 2025.

